#  

**DOI:** 10.1002/pul2.12112

**Published:** 2022-08-17

Residence at moderately high altitude and its relationship with WHO Group 1 pulmonary arterial hypertension symptom severity and clinical characteristics: the Pulmonary Hypertension Association Registry.

Shoaib Fakhri, Kelly Hannon, Kelly Moulden, Ryan Peterson, Peter Hountras, Todd Bull, James Maloney, Teresa De Marco, Dunbar Ivy, Thenappan Thenappan, Jeffrey S. Sager, John J. Ryan, Sula Mazimba, Russel Hirsch, Murali Chakinala, Oksana Shlobin, Matthew Lammi, Dianne Zwicke, Jeffrey Robinson, Raymond L. Benza, James Klinger, Daniel Grinnan, Stephen Mathai and David Badesch; on behalf of the PHAR Investigators.

Pulmonary Circulation, 10 (4), 2020. DOI: https://doi.org/10.1177/2045894020964342.

An error was reported to us by the PHAR registry in coding that has impacted data interpretation on one of our quality of life metrics, the SF‐12.

The coding error failed to reverse code responses for four of the twelve SF‐12 questions. All twelve questions are used (in different proportions) to calculate both the mental and physical SF‐12 scores. The reverse coding error impacts both the physical and mental SF‐12 scores, although in different proportions.

Participant responses to questions are coded on a scale of one to five, where one is the worst outcome, and five is the best outcome. The four questions below should have been reverse coded to follow the same scale.

**Table:** 

Question Number	Text	Responses
SF‐12 Q1	In general, would you say your health is:	1. Excellent 2. Very Good 3. Good 4. Fair 5. Poor
SF‐12 Q8	During the past 4 weeks, how much did pain interfere with your normal work (including both work outside the home and housework)?	1. Not at all 2. A little bit 3. Moderately 4. Quite a bit 5. Extremely
SF‐12 Q9	How much of the time during the past 4 weeks…	1. All of the time 2. Most of the time 3. Some of the time 4. A little of the time 5. None of the time
	Have you felt calm and peaceful	
SF‐12 Q10	How much of the time during the past 4 weeks…	1. All of the time 2. Most of the time 3. Some of the time 4. A little of the time 5. None of the time
	Did you have a lot of energy	

Participant answers are preserved and accurate; however, the coding of the data for certain questions was written in error. We have rerun our analysis.

The results are qualitatively the same as in our published paper (we find no effect of elevation on physical health score, either marginally or after adjusting for covariates). The p‐value is lower but still insignificant.

Since multiple imputation is used for missing data, the slight change in the SF‐12 score impacts imputed values for other covariates, which then causes very slight changes to all the other numbers in the report. Again, no qualitative differences.

A PHAR data update was released post‐publication. Analysis was re‐run on the updated data, results/conclusions did not substantively change.

We apologize for the error.

